# *Campylobacter jejuni* extracellular vesicles harboring cytolethal distending toxin bind host cell glycans and induce cell cycle arrest in host cells

**DOI:** 10.1128/spectrum.03232-23

**Published:** 2024-02-06

**Authors:** Lena Hoang My Le, Bassam Elgamoudi, Nina Colon, Angus Cramond, Frederic Poly, Le Ying, Victoria Korolik, Richard L. Ferrero

**Affiliations:** 1 Centre for Innate Immunity and Infectious Diseases, Hudson Institute of Medical Research, Victoria, Australia; 2 Department of Microbiology, Biomedicine Discovery Institute, Monash University, Victoria, Australia; 3 Institute for Glycomics, Griffith University, Gold Coast, Queensland, Australia; 4 Enteric Diseases Department, Naval Medical Research Centre, Silver Spring, Maryland, USA; 5 Department of Molecular and Translational Sciences, Monash University, Victoria, Australia; Universidad Andres Bello, Santiago, Chile

**Keywords:** outer membrane vesicles, OMVs, *Campylobacter jejuni*, campylobacter, toxins, cytolethal distending toxin, host-pathogen interactions, enteric pathogens, mucosal pathogens

## Abstract

Cytolethal distending toxins (CDTs) are released by Gram-negative pathogens into the extracellular medium as free toxin or associated with extracellular vesicles (EVs), commonly known as outer membrane vesicles (OMVs). CDT production by the gastrointestinal pathogen *Campylobacter jejuni* has been implicated in colorectal tumorigenesis. Despite CDT being a major virulence factor for *C. jejuni*, little is known about the EV-associated form of this toxin. To address this point, C. *jejuni* mutants lacking each of the three CDT subunits (A, B, and C) were generated. *C. jejuni cdtA, cdtB,* and *cdtC* bacteria released EVs in similar numbers and sizes to wild-type bacteria, ranging from 5 to 530 nm (mean ± SEM = 118 ±6.9 nm). As the CdtAC subunits mediate toxin binding to host cells, we performed “surface shearing” experiments, in which EVs were treated with proteinase K and incubated with host cells. These experiments indicated that CDT subunits are internal to EVs and that surface proteins are probably not involved in EV-host cell interactions. Furthermore, glycan array studies demonstrated that EVs bind complex host cell glycans and share receptor binding specificities with *C. jejuni* bacteria for fucosyl GM1 ganglioside, P1 blood group antigen, sialyl, and sulfated Lewis^x^. Finally, we show that EVs from *C. jejuni* WT but not mutant bacteria induce cell cycle arrest in epithelial cells. In conclusion, we propose that EVs are an important mechanism for CDT release by *C. jejuni* and are likely to play a significant role in toxin delivery to host cells.

**IMPORTANCE:**

*Campylobacter jejuni* is the leading cause of foodborne gastroenteritis in humans worldwide and a significant cause of childhood mortality due to diarrheal disease in developing countries. A major factor by which *C. jejuni* causes disease is a toxin, called cytolethal distending toxin (CDT). The biology of this toxin, however, is poorly understood. In this study, we report that *C. jejuni* CDT is protected within membrane blebs, known as extracellular vesicles (EVs), released by the bacterium. We showed that proteins on the surfaces of EVs are not required for EV uptake by host cells. Furthermore, we identified several sugar receptors that may be required for EV binding to host cells. By studying the EV-associated form of *C. jejuni* CDT, we will gain a greater understanding of how *C. jejuni* intoxicates host cells and how EV-associated CDT may be used in various therapeutic applications, including as anti-tumor therapies.

## INTRODUCTION

Cytolethal distending toxins (CDTs) are a family of heat-labile genotoxins produced by Gram-negative pathogens, such as *Aggregatibacter actinomycetemcomitans*, *Campylobacter jejuni*, *Escherichia coli*, *Haemophilus ducreyi,* and *Helicobacter hepaticus* (1). These AB_2_ toxins share a tripartite structure comprising CdtA, CdtB, and CdtC proteins (2, 3). CdtA and CdtC are the cell surface-binding moieties that facilitate the translocation of the active CdtB subunit into host cells (4). CdtB is the most functional conserved with type 1 deoxyribonuclease activity and induces DNA damage, leading to cell cycle arrest at the G1/G2 phase in epithelial cells and apoptosis in sensitive cells (5, 6). CdtB can also function as a phosphatidylinositol-3,4,5 triphosphate (PIP3) phosphatase to disrupt PI-3K signaling, resulting in pro-inflammatory cytokine production (7).

Despite sharing a functional commonality with the CDTs produced by other Gram-negative pathogens, that produced by *C. jejuni* (Cj-CDT) is reported to be the most divergent toxin and exhibits exceptional host cell specificity (8). *C. jejuni* is a major causative agent of bacterial gastroenteritis and childhood mortality due to diarrheal disease worldwide (9, 10). Campylobacteriosis tends to be self-limiting but can also lead to inflammatory bowel disease (11), colorectal cancer (12), reactive arthritis (13), septicemia (14), and severe neurological sequelae, such as Guillain-Barré and Fisher syndromes (10). Despite the significant medical impact of *C. jejuni* infection, little is still known about its pathogenesis. As the majority of *C. jejuni* strains tested were found to produce active CDT (15), this toxin was suggested to be an important virulence factor for the bacterium. Cj-CDT promotes a pro-inflammatory environment with uncontrolled bacterial proliferation and impaired renewal of intestinal cells in the host (16). Recent studies suggested that CDT production in *C. jejuni* promotes the initiation of colorectal cancer by inducing changes in the microbial composition and transcriptomic responses (17).

In addition to being membrane-bound and secreted as a soluble toxin, CDTs can also be associated with extracellular vesicles (EVs) (18), often referred to as outer membrane vesicles (OMVs). EVs are spherical nanostructures (20–400 nm) ubiquitously released during all growth stages of bacteria and have many important functions, including their ability to deliver a mixture of virulence determinants into host cells (19). Similar to the whole bacteria, *C. jejuni* EVs were observed to possess cytotoxic activity and induce interleukin-8 (IL-8), interleukin-6 (IL-6), tumor necrosis factor (TNF), and beta-defensin-3 (hBD-3) production in T84 intestinal epithelial cells (20). In addition to delivering virulence determinants to host cells, *C. jejuni* EVs can enhance bacterial adhesion and invasion of intestinal and colonic epithelial cells (21, 22). For example, *C. jejuni* EVs harbor proteins (e.g., HtrA, Cj0511, Cj1365c) with proteolytic activity that can cleave E-cadherin and occludin, which are components of the adherens and tight junctions, respectively (23). Thus, *C. jejuni* EVs can facilitate the colonization of bacteria to host cells, as well as modulate their responses to promote bacterial survival *in vivo*.

One study reported that although Cj-CDT can be recovered in a secreted form from culture supernatants, most of the toxin is associated with EVs, highlighting the importance of EVs as a likely delivery mechanism of CDTs to host cells (18). Nevertheless, much of the research to date on Cj-CDT has used either recombinant CDT subunits or crude bacterial lysates containing CDTs (4, 24–26). The current study aimed to therefore study EV-associated CDT produced by *C. jejuni* and determine its effect on host cells. To accomplish this, we first generated *C. jejuni cdtA*, *cdtB,* and *cdtC* mutants. The EVs from the *C. jejuni* wild type (WT) and mutant strains were isolated and characterized. For the first time, we provide evidence that all three CDT subunits are internal to EVs, suggesting that CdtAC is unlikely to mediate vesicle binding to host cells. By performing glycan array studies, we showed that *C. jejuni* EVs from three different strains had poor specificities for mono- and di-saccharides, instead preferentially binding complex glycans and exhibiting shared receptor specificities with the whole bacteria for several blood group antigens and gangliosides. Finally, we show that *C. jejuni* EVs harboring CDT holotoxin-induced cell cycle arrest and cell distension of epithelial cells. In conclusion, we have performed the first detailed characterization of CDT associated with *C. jejuni* EVs and identified potential glycan receptors for EV entry into host cells. We propose that EVs represent a major mechanism for CDT release by bacteria and are likely to play a significant role in the delivery of these toxins to host cells.

## MATERIALS AND METHODS

### Cell line, bacterial strains, and growth conditions

HeLa cells were maintained in complete Roswell Park Memorial Institute (RPMI) medium (Gibco) supplemented with 10% (vol/vol) fetal bovine serum (Gibco), 1% (vol/vol) L-glutamine (Gibco), and 1% (vol/vol) penicillin/streptomycin (Gibco). *C. jejuni* strains 11168-O (27), 81-116 (27), and 81-176 (28) were routinely cultured on Muller Hinton Agar (MHA; Thermo Fisher Scientific) or broth containing Skirrow’s selective supplement as previously described (29). The medium was supplemented with 10 µg/mL tetracycline (Sigma-Aldrich) and/or 20 µg/mL kanamycin (Sigma-Aldrich), as required. Bacteria were incubated at 37°C under microaerobic conditions (Campygen, Oxoid, Thermo Fisher Scientific). For plasmid construction and selection of transformants, chemically competent *E. coli* (XL-1 blue) were grown at 37°C in plain Luria-Bertani (LB) agar or broth supplemented with ampicillin (100 µg/mL) or kanamycin (20 µg/mL).

### Construction of *C. jejuni cdt* mutants

*C. jejuni cdtA*, *cdtB,* and *cdtC* mutants were generated previously using a promoter-driven kanamycin cassette (PD Km^R^) (28). To generate a *C. jejuni cdtA* mutant harboring a promoter-less Km^R^ cassette (PL Km^R^), the *cdtA* gene was PCR amplified from *C. jejuni* 81-116 genomic DNA using cdtA-For and cdtA-Rev primers (Table S1). The 728 base-pair fragment was inserted into pGEM-T-Easy Vector (Promega), as per the manufacturer’s instructions. Inverse PCR was performed with KOD hot start DNA polymerase (Sigma-Aldrich), using primers containing *BamHI* and *KnpI* restriction sites (Table S1), and ligated to a non-polar Km^R^ cassette (aphA-3), digested from pUC18K-2 (30, 31). A Purelink HiPure plasmid filter MidiPrep kit (Thermo Fisher Scientific) was used to purify the constructs to be electro-transformed into *C. jejuni* strains 11168-0, 81-116, and 81-176, as described previously (32), and selected on MHA containing Km (20 µg/mL). Km^R^ transformants were verified by PCR and Sanger sequencing using primers for the cassette (H17& H50) and the *cdtA* gene. Genomic DNA was extracted using the PureLink Genomic DNA mini kit (Thermo Fisher Scientific). Polymerase chain reactions (PCR) were as follows: 95°C for 5 min, followed by 35 cycles of 94°C for 30 sec, 57-59°C for 1 min, 72°C for 2 min, and a final extension step of 72°C for 2 min.

### Quantitative PCR (qPCR) analysis

RNA was extracted from *C. jejuni* grown to mid-exponential phase in MH broth (25 mL) using TRIzol reagent (Thermo Fisher Scientific), as per the manufacturer’s instructions. cDNA was generated from 1000 ng of RNA using the Tetro cDNA synthesis kit (Bioline). qPCR reactions consisted of 4 µL of diluted synthesized cDNA (1:4), 5 µL SYBR Green qPCR MasterMix (Thermo Fisher Scientific), and 1 µL of primer for the gene of interest (Table 1). qPCR assays were performed using a QuantStudio 6 Flex Real-Time PCR machine (Thermo Fisher Scientific) and the following program: 50°C, 2 min followed by 95°C, 10 min then 45 cycles of amplification (95°C, 15 s; 60°C, 1 min) and a melt curve stage (95°C, 15 s; 60°C, 1 min; 95°C, 15 s). Relative gene expression levels in samples were determined using the delta-delta Ct method with Ct values normalized to those of the 16S rRNA gene.

**TABLE 1:** The glycans bound by bacteria and EVs of *C. jejuni* strains 81-116, 81-116, and 11168-O^*a*^

Glycan type	Strains					
	81-116		81-176		11168-O	
	Bacteria	EVs	Bacteria	EVs	Bacteria	EVs
Monosaccharides & Di-saccharides						
Fucα-sp3	+	-	+	-	+	-
Galα-sp3	+	-	+	-	+	-
β-GlcNAc	+	-	+	-	+	-
Manα-sp3	+	-	+	-	+	-
Neu5Acα-sp3	+	-	+	-	+	-
β-Glc6P	+	-	-	-		-
GalNAcα-sp3	+	-	-	+	+	-
GlcNAcβ1–6GalNAcα-sp3	+	-	+	-	+	-
Galβ1–2Galβ-sp3	+	-	+	+	+	-
GalNAcα1–3GalNAcα-sp3	+	-	+	-	+	-
Manα1–3Manβ-sp4	+	-	-	-	+	-
Fucα1–4GlcNAcβ-sp3	+	-	+	-	+	-
Neu5Gcα2–3Gal-sp3	+	-	+	-	+	-
Tri-saccharide and tetra-saccharides						
Galα1–4Galβ1–4Glcβ-sp2 (P1)	+	+	+	+	+	+
Galβ1–3(Fucα1–4)GlcNAc (Le^a^)	+	+	-	-	+	+
Fucα1–3(3-O-Su-Galβ1–4)GlcNAcβ-sp3 (Su-Le^x^)	+	+	+	+	+	+
Fucα1–2Galβ1–3GalNAcα-sp3	+	-	+	-	-	-
Fucα1–3(Galβ1–4)GlcNAcβ-sp3	-	-	+	-	-	-
GlcNAcβ1–4GlcNAcβ1–4GlcNAcβ-sp4	-	-	+	+	-	-
Neu5Acα2–3Galβ1–4GlcNAc	+	+	+	+	+	+
Neu5Acα2–3Galβ1–4Glcβ-sp4	+	-	+	-	-	-
Neu5Acα2–3Galβ1–4-(6-O-Su)GlcNAcβ-sp3	-	-	+	-	+	-
Neu5Acα2–6Galβ1–4Glcβ-sp2	+	-	+	-	+	-
Neu5Acα2–6Galβ1–3GlcNAc-sp3	+	-	-	-	+	-
Galβ1–3GalNAcβ1–4Galβ1–4Glcβ-sp3 (Asialo-GM1)	-	-	+	+	-	-
GalNAcβ1–3Galα1–4Galβ1–4Glcβ-sp3	+	-	+	-	+	-
Fucα1–3(Neu5Acα2–3Galβ1–4)GlcNAcβ-sp3	+	-	+	-	+	-
Neu5Acα2–3Galβ1–3(Fucα1–4)GlcNAc (Sia-Le^a^)	+	-	+	+	+	+
Complex Glycan						
GalNAcα1–3(Fucα1–2)Galβ1–3GalNAcβ1–3Gal (Blood group A antigen pentose type 4)	+	+	+	+	-	-
Galα1–3(Fucα1–2)Galβ1–4(Fucα1–3)Glc (Blood Group B pentasaccharide)	-	-	-	-	+	+
GalNAcα1–3(Fucα1–2)Galβ1–4(Fucα1–3)Glc (Blood group A pentasaccharide)	-	-	-	-	+	+
Fucα1–2Galβ1–3GalNAcβ1–4(Neu5Acα2–3)Galβ1–4Glc (fucosyl GM1)	+	+	+	+	+	+
Neu5Acα2–8Neu5Acα2–3Galβ1–4Glc (GD3)	+	+	+	+	-	-
Fucα1–2Galβ1–3(Fucα1–4)GlcNAcβ1–3Gal (Le^b^)	+	-	+	-	+	-
Galβ1–3GalNAcβ1–4(Neu5Acα2–8Neu5Acα2–8 Neu5Acα2–3)Galβ1–4Glc (GT1c)	-	-	-	-	+	+
Neu5Acα2–8Neu5Acα2–3Galβ1–3GalNAcβ1–4(Neu5Acα2–3)Galβ1–4Glc (GT1a)	+	+	-	-	+	+
Neu5Acα2–3Galβ1–3GalNAcβ1–4(Neu5Acα2–3)Galβ1–4Glc (GD1a)	-	-	-	+	+	+
GlcAβ1–3GlcNAcβ1–4)n (*n* = 4)	+	-	+	-	+	-

^*a*^ Glycans are clustered into classes based on their respective terminal sugars. Fuc, fucose; Man, mannose; Gal, galactose; GalNAc, 2′-N-acetyl galactosamine; Glc, glucose; GlcNAc, 2′-N-acetyl glucosamine; Neu5Acα2, sialylated GlcA; D-Glucuronic acid; sp3/4, the linker tethering the glycans to the slides. Complex glycans: Lewis (Le) antigens, Le^a^, Le^b^, Le^x^; P_1_ antigen; asialo GM1 ganglioside (Asialo-GM1); and ganglioside sugars, GD3, GT1a, GT3. +, indicates binding; -, indicates no binding. Data are presented for *n* = 2–3 biological replicates. The full data set is presented in Table S3.

### EV isolation

*C. jejuni* EVs were isolated as described (33) with some modifications. In brief, *C. jejuni* strains were inoculated to an optical density (OD_600_) of 0.1 in depleted Brain Heart Infusion (D-BHI; BD Bacto) broth containing Skirrow’s selective supplement and tetracycline (10 µg/mL) or kanamycin (20 µg/mL), as required. Cultures were incubated for 17 hours (mid-exponential phase) under microaerobic conditions at 37°C while shaking at 120 rpm. Bacteria were removed from cultures by low-speed centrifugation (4000 × *g* for 20 min at 4°C), followed by filtration through 0.22 μm pore membranes. Cell-free cultures were subjected to ultracentrifugation (100,000 × *g* for 2 hours at 4°C) and the resulting concentrated EVs were further washed three times, each with 3 mL of PBS, using 100,000 MWCO Amicon filters (Merck). EV preparations were used immediately or stored at −20°C.

### Electron microscopy

EVs (100 µg/mL) were visualized by negative staining on the FEI Tecnai Spirit electron microscope (Fei Company), as described previously (33). Images were randomly selected across the grids at 42,000 x magnification. Three fields were imaged for each biological replicate of EVs.

### Nanoparticle tracking analysis

The size distributions and numbers of particles were determined by nanoparticle tracking analysis (NTA) using a ZetaView instrument (Particle Matrix). Measurements were performed using a 488 nm laser and CMOS camera by scanning 11 cell positions and capturing 60 frames per position at 24.5 degrees with a camera sensitivity of 80, shutter speed of 100, and autofocus and automatic scattering intensity. Analysis was performed using ZetaView Software version 8.05.14 SP7 with a minimum brightness of 30, a maximum brightness of 255, a minimum area of 10, a maximum area of 1,000, and a minimum trace length of 15.

### Anti-EV serum production

Rabbit polyclonal antiserum to *C*. *jejuni* EVs was generated by the antibody facility at the Walter and Eliza Hall Institute of Medical Research (WEHI), Melbourne, Australia. To ensure broad reactivity of the antiserum, EVs were isolated from three *C. jejuni* strains (81-116, 81-176, and 11168-O). Rabbits were administered EVs (200 µg) with complete Freund’s adjuvant (CFA) at day one, then with incomplete Freund’s adjuvant at days 28 and 56.

### Immunoblot analysis

Bacterial lysates and EV samples were resuspended in 4 x Laemmli sample buffer (Bio-Rad) and heated at 70°C for 10 min before loading (7.5 µg) on NuPAGE 4%-12% Bis-Tris 1.5 mm gels (Thermo Fisher Scientific). After electrophoresis, the proteins were transferred onto 0.45 µm immobilon-PVDF membranes (Merck) at 100 V for 70 min. Membranes were stained with Ponceau S (Sigma-Aldrich) to examine transfer efficiency before blocking with 5% skim milk in TBS-Tween 20 (0.05% vol/vol) for 1 h. Blots were incubated overnight at 4°C with primary antibodies (Table S2) that had been diluted in 1% skim milk in TBST-Tween 20 (0.05% vol/vol*).* Antibodies to *Helicobacter pylori* GroEL (also known as HspB) were used to detect GroEL from *C. jejuni*, as the proteins from these two bacteria share 76% identity at the protein level. For RpoD detection, *E. coli* anti-RNA sigma 70 antibody (clone 2G10; Table S2) was used as the epitope mapped to amino acids 470–486 (34), shared by *C. jejuni* RpoD. Blots were washed three times with TBS-Tween 20 (0.05% vol/vol) and incubated for 2 h at room temperature with either rabbit anti-mouse IgG horseradish peroxidase (HRP; 31450, Thermo Fisher Scientific) or goat anti-rabbit IgG HRP (31460, Thermo Fisher Scientific) diluted 1:2,000 in 1% skim milk. For biotin-labeled proteins, blots were incubated with streptavidin-HRP (1:1,000) for 1 h at room temperature before three washes with TBS-Tween. Membranes were developed with Amersham ECL immunoblotting reagent (GE Healthcare) and imaged using an Amersham Imager 680 reagent (GE Healthcare).

### Proteinase K treatment of EVs

EV surface proteins were removed by proteinase K treatment as described previously (35) with modification. In brief, EVs were diluted to 450 µg/mL of protein and incubated with 20 µg/mL proteinase K (Thermo Fisher Scientific) for 1 hour at 37°C in the presence or absence of 1% (w/v) SDS. Proteinase activity was inhibited by treatment with 5 mM phenylmethylsulphonyl fluoride (Thermo Fisher Scientific) for 10 min at room temperature. The DSB-X biotin labeling kit (Thermo Fisher Scientific) was used to label surface EV proteins, as per the manufacturer’s instructions.

### Cell cycle analysis by flow cytometry

HeLa cells were seeded in 6-well plates (2 × 10^5^ cells/mL) and exposed to 10 µg (5 µg/mL) of *C. jejuni* EVs for 24 hours. After washing with PBS, cells were treated with 1 mL TrypLe express enzyme (Thermo Fisher Scientific) for 3 min at 37°C. Complete RPMI (4 mL) was added to the wells before cells were pelleted (300 × *g*, 5 min) and resuspended in 0.5 mL PBS. Cells were fixed with 4.5 mL cold 70% ethanol for 4 days at −20°C before being harvested by centrifugation and rehydrated with 5 mL cold PBS for 1 min. Following centrifugation, cells were resuspended in 300 µL propidium iodide (PI) staining buffer (500 µg/mL PI [Biolegend], 0.1% vol/vol Triton X [Sigma-Aldrich], 0.2 mg/mL DNase-free RNase A [Sigma-Aldrich]) and incubated in the dark at room temperature for 30 min. The cells were then analyzed using a BD LSR Fortessa X-20 flow cytometer (BD Biosciences), followed by FlowJo software (Version 10; Treestar, Inc).

### Immunofluorescence staining

HeLa cells were seeded in 96-well glass bottom plates (1 × 10^4^ cells/mL; Corning) and stimulated with *C. jejuni* EVs (5 µg/mL) for 48 hours. After washing in PBS, cells were stained with 100 µL of 1 x CellMask Green (Thermo Fisher Scientific) and Hoechst (1:2,000; Thermo Fisher Scientific). Cells were fixed with 4% paraformaldehyde (PFA) for 5 min at 37°C and washed three times with PBS. Imaging was performed with an FV1200 Confocal Microscope (Evident Australia) using a 20×, 0.75 NA objective with excitation at 405 nm for Hoechst and 488 nm for CellMask Green. Acquisition parameters were maintained at consistent settings for all images acquired. Cell cytoplasm and nucleus areas were quantified using the cell function on Imaris software (Bitplane, Oxford instruments). The algorithm used was for nucleus and cell detection. Nuclei were detected using a smooth filter width of 1.40 µm. Cell body detection used a cell smooth filter width of 1.50 µm, expanded on the nucleus, and assumed one nucleus per cell.

### Glycan array analysis

Glycan arrays were printed as described previously (36, 37). Briefly, ~367 distinct glycans were spotted and covalently linked to epoxy-coated glass slides (ArrayIt). The slides were blocked with 0.1% (w/v) BSA in PBS for 5 min, then dried by centrifugation at 200 × *g* for 4 min before incubation with *C. jejuni* bacteria or EVs. Bacteria harvested in PBS were fixed with 2.5% (wt/vol) formaldehyde for 30 min, then labeled with 10 µM CellTrace BODIPY TR Methyl ester lipophilic stain (Thermo Fisher Scientific) for 30 min. Excess dye was removed by three washes with PBS before resuspension of cells in array PBS (containing 2 mM MgCl_2_ and 2 mM CaCl_2_) to an optical density (OD_600_) = 0.1. EVs were labeled with 10 µM CellTrace BODIPY TR Methyl ester lipophilic stain for 30 min and washed three times in PBS. Labeled bacteria and EVs were added to array slides and incubated for 30 min in the dark at room temperature. Slides were washed three times with array PBS and dried *via* centrifugation at 200 × *g* for 4 min. The array slides were scanned and analyzed using the ScanArray Express software program (Perkin Elmer). Two biological replicates were performed. Glycan binding was classified as positive if the relative fluorescence unit value was greater than one-fold above the mean background (defined as the average background of negative control spots plus three standard deviations) and was statistically significant (*P* < 0.05, student *t*-test) (36, 37).

### Statistical analysis

GraphPad Prism v.9.4.1 (GraphPad Software) was used for statistical analysis of the data and its graphical representation. qPCR results were analyzed by either the Mann-Whitney U or Kruskal-Wallis tests. Cell cycle analysis and cell immunofluorescence results were compared by one-way ANOVA followed by Tukey multiple comparison test. Values of *P <* 0.05 were considered statistically significant.

## RESULTS

### *C. jejuni* WT and *cdt* isogenic mutants produce EVs with similar morphologies and particle size distributions

**Fig 1:**
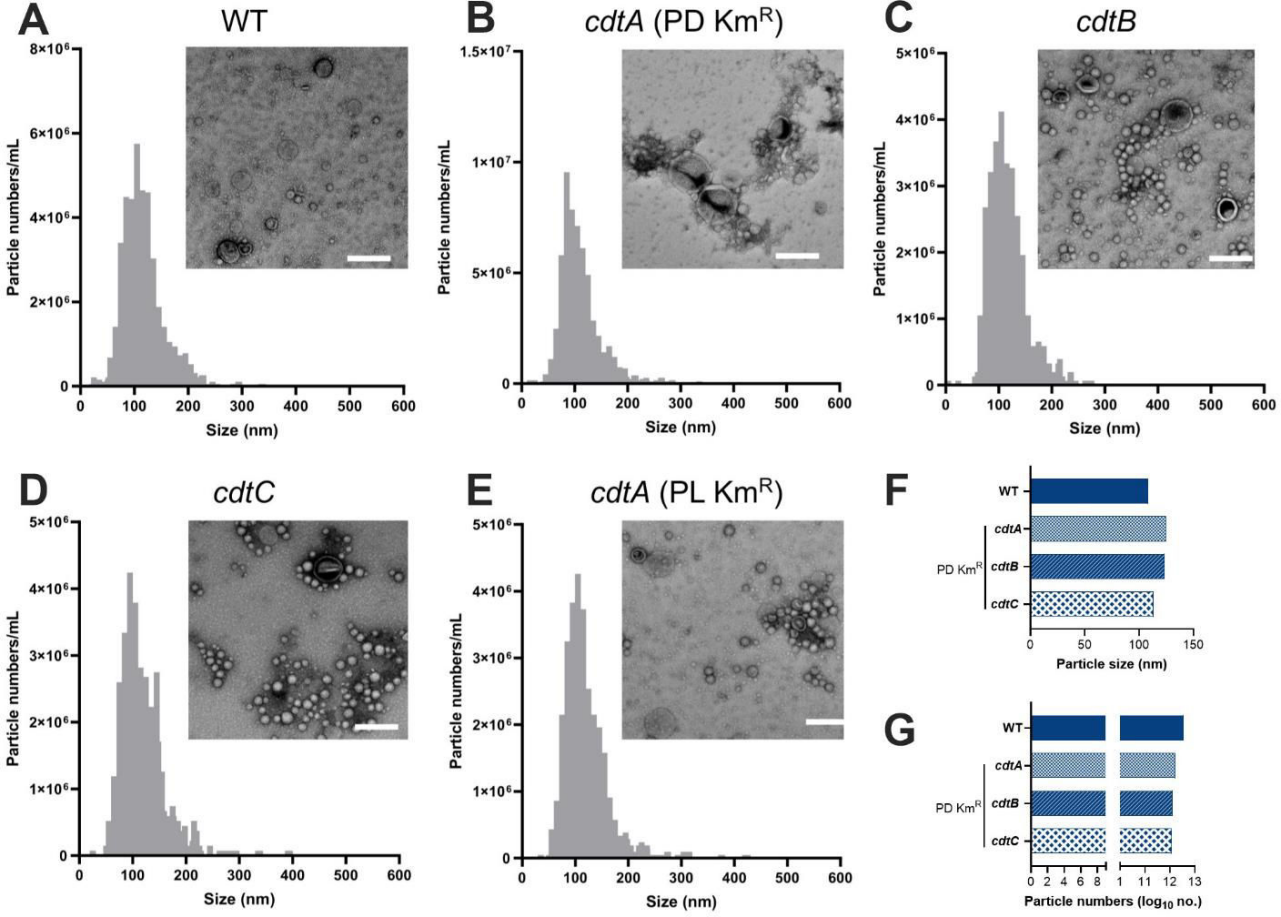
*C. jejuni* WT and *cdt* bacteria produce EVs with similar morphologies and particle size distributions. EVs from *C. jejuni* 81-176 WT and *cdt* mutants were analyzed by NTA and TEM. (**A–E**) Particle size distributions and numbers of isolated EVs (diluted 1:4,000–1:20,000). The top right image in each panel shows isolated EVs analyzed by TEM (42,000× magnification). Scale bar = 200 µm. (**F**) Mean particle size and (**G**) numbers of particles of the isolated EVs. Images are representative of three fields from one independent experiment.

To study the role of EV-associated CDT in host cell interactions, we generated *C. jejuni cdtA*, *cdtB,* and *cdtC* mutants on the 81–176 strain background, using either PD Km^R^ or PL Km^R^ cassettes. EVs from *C. jejuni* WT and isogenic *cdt* mutants were characterized by TEM and NTA to ensure that the mutagenesis procedure had not had an impact on their production or physical characteristics. TEM analysis confirmed that all the EV preparations contained particles with similar morphologies, consisting of heterogeneous populations of spherical structures, surrounded by double membrane layers, including some concave forms, which are characteristic of both procaryotic and eucaryotic EVs (19) (Fig. 1E). The EV preparations also had similar particle numbers and size distributions, ranging from 5 to 530 nm (mean ± SEM = 118 ±6.9 nm) (Fig. 1A through G). Together, these data indicate that mutagenesis of the *cdt* genes had no significant impact on EV production or their physical characteristics.

### Cj-CDT is mainly located within EVs and induces cell distension in epithelial cells in the absence of surface proteins on EVs

**Fig 2:**
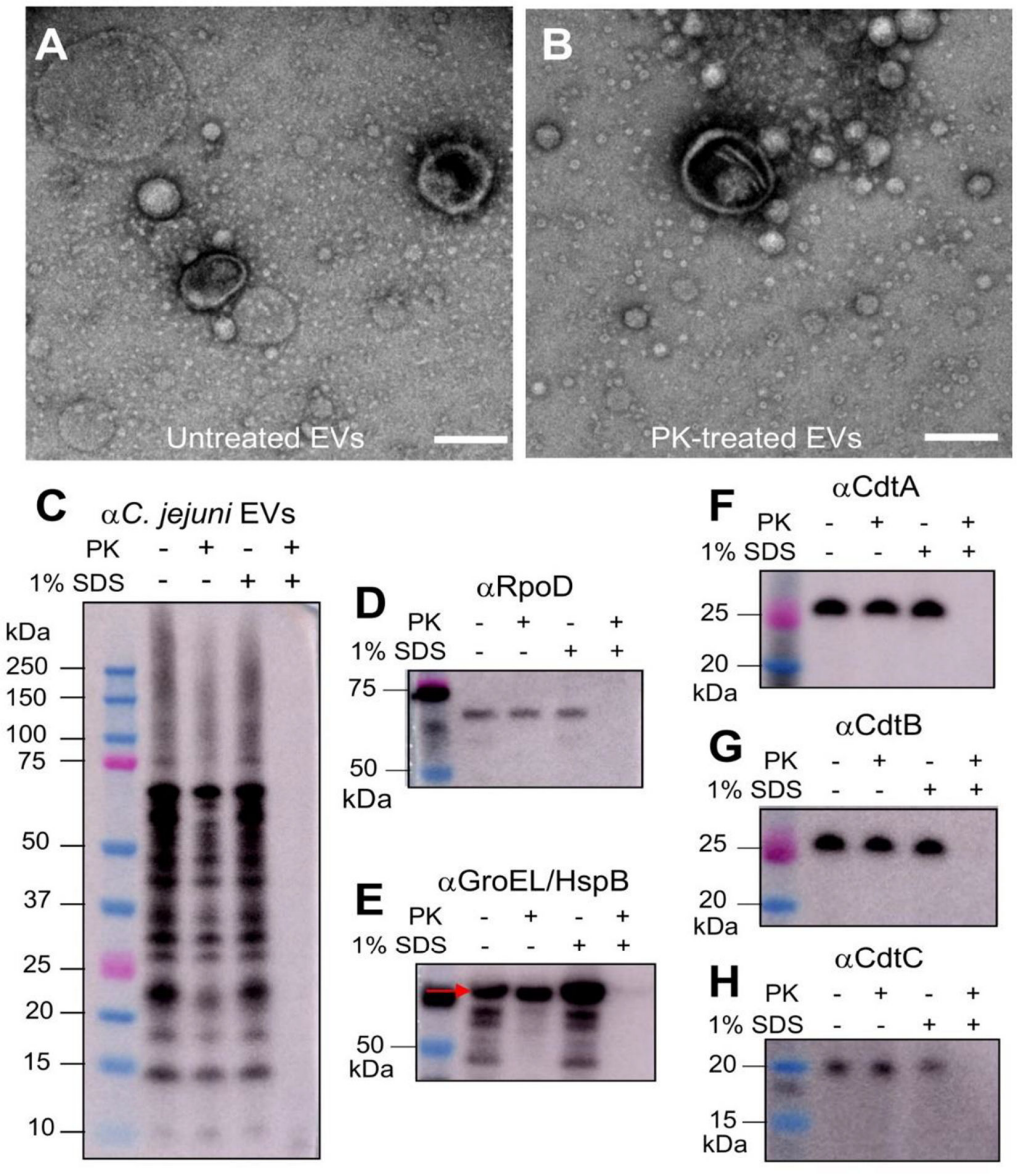
*C. jejuni* CDT subunits are mainly located within EVs. Surface-accessible proteins on *C. jejuni* 81-176 EVs were digested using proteinase K (PK) in the presence or absence of 1% SDS for 1 hour at 37°C. (**A, B**) TEM images of untreated and PK-treated EVs (42,000× magnification). Scale bar = 200 µm. Images are representative of three fields. EVs were analyzed by immunoblotting with antisera against (**C**) *C. jejuni* EVs, (**D**) RpoD, (**E**) GroEL (also known as HspB), (**F**) CdtA, (**G**) CdtB, and (**H**) CdtC subunits. (**C, F–H**) immunoblots are representative of four independent experiments, whereas (**D, E**) Immunoblots are from one independent experiment.

Certain bacterial toxins and proteins, which are associated with EVs, were found to enhance the binding of EVs with host cells (38–40). It is unclear, however, whether one or more of the Cj-CDT subunits, particularly the putative host cell-binding moieties CdtAC, may be surface exposed on EVs and therefore mediate their uptake into host cells. We determined whether any of the CDT subunits may be externally exposed on EVs using a “surface shearing” method (35), whereby proteinase K (PK) was used to remove surface-accessible proteins on these particles. CDT proteins that may be internal to the EVs were released by treatment with 1% SDS. To ensure that the PK treatment did not cause lysis of EVs, PK-treated EVs were examined by TEM, which showed that the EVs remained intact after this treatment (Fig. 2A and B). Immunoblotting of the EVs with anti-*C. jejuni* EV serum showed that PK was able to efficiently digest surface proteins on EVs (Fig. 2C) as well as those located within EVs that had been treated with 1% SDS (Fig. 2C). To confirm the ability of PK to remove surface EV proteins, vesicles were labeled with DSB-X biotin before PK treatment. Immunoblotting of PK-treated EVs with streptavidin-HRP showed that fewer biotin-labeled proteins were present after PK treatment (Fig. S1), suggesting the successful removal of surface EV proteins by PK. As controls, we also used RpoD and GroEL, which were reported to be cytoplasmic and associated with outer membranes in bacteria, respectively (41). As expected, RpoD levels were unaffected by PK treatment alone (Fig. 2D). Surprisingly, however, GroEL was also unaffected by this treatment (Fig. 2E). We speculate that GroEL may be embedded in the EV membrane and not surface exposed, as increased amounts of GroEL were detected in the presence of 1% SDS (Fig. 2E). Importantly, the PK treatment appeared to have little effect on CdtA, CdtB, or CdtC levels in the EV preparations, when compared with untreated EVs (Fig. 2F through H), suggesting that all three subunits are mainly localized within EVs.

**Fig 3:**
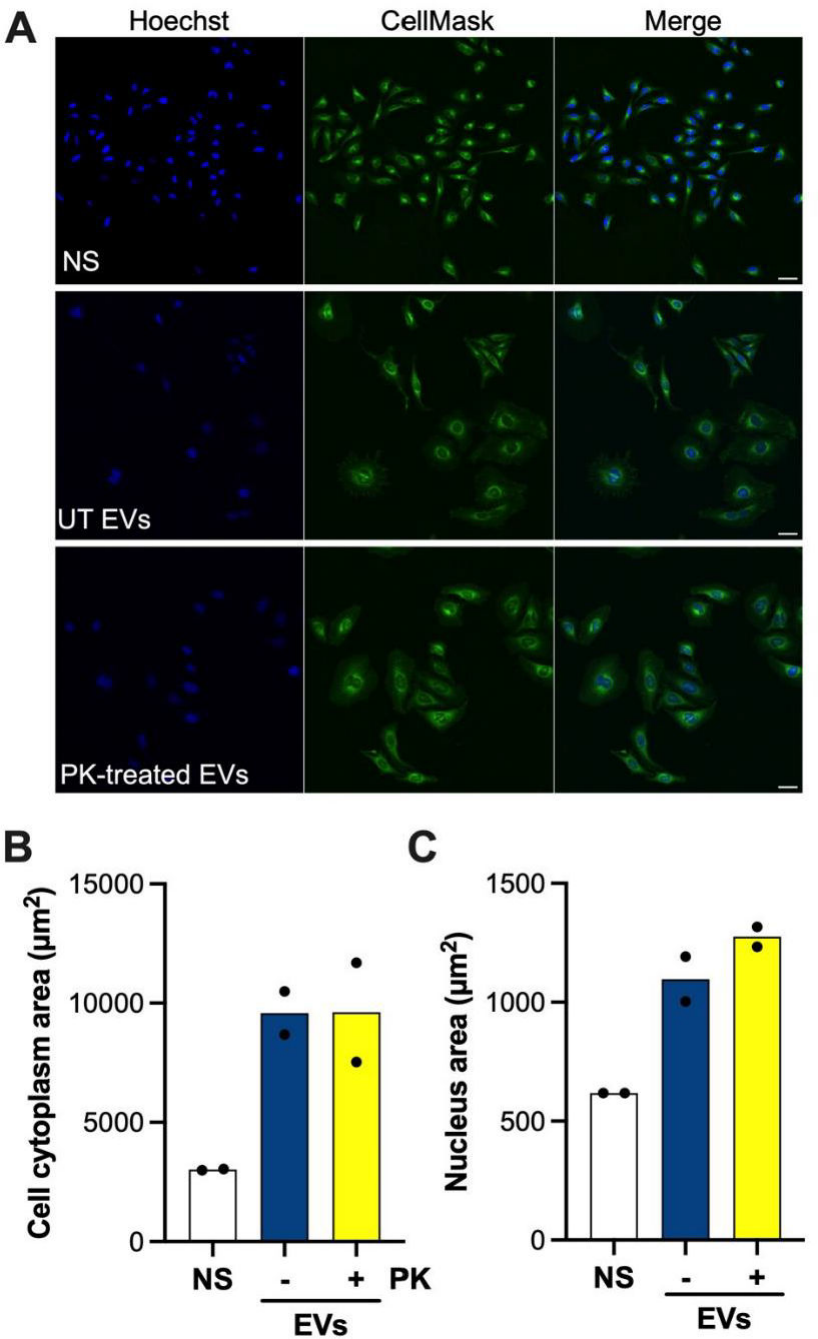
*C. jejuni* CDT does not require surface proteins on the EVs to induce cell distension in HeLa cells. (**A**) HeLa cells were incubated with either untreated (UT) or PK-treated EVs (5 µg/mL) for 48 hours before staining with Hoescht 33342 (nucleus) and CellMask Green (plasma membrane) dyes. Control cells were not exposed to EVs (not stimulated, NS). Images were acquired on an Olympus FV1200 Confocal Microscope (20× magnification). Scale bar = 50 µm. Images are representative of two independent experiments. (**B**) Cytoplasmic and (**C**) nuclear areas of cells were quantified using Imaris software. Each data point represents the mean value for a biological replicate calculated from 3 to 4 individual fields.

Next, we sought to determine whether surface proteins on *C. jejuni* EVs may be required for cell entry by EV-associated Cj-CDT. Thus, EVs were either PK-treated or, as a control, left untreated. We then measured cell distension, which occurs during cell intoxication by *C. jejuni* CdtB (42), as a surrogate for cell entry in HeLa cells that had been incubated with the untreated or PK-treated EVs. The absence of surface proteins on EVs appeared to have no effect on their ability to induce CdtB-mediated cell distension (Fig. 3A). This was confirmed by quantification of the cell cytoplasm and nucleus areas in the EV-treated cells (Fig. 3B and C). Taken together, the results demonstrated that biologically active *C. jejuni* Cj-CDT is localized mainly within EVs and, moreover, suggest that protein ligands on the EV surface are not required for uptake in host cells.

### *C. jejuni* EVs preferentially bind to complex host cell glycans

The mechanism of uptake into host cells for *C. jejuni* EVs, as well as the cellular receptors involved, has yet to be elucidated. As host glycans were reported to be crucial for *C. jejuni* bacterial interactions with host cells (36, 37), we performed glycan array analyses with EVs from three different *C. jejuni* strains, as well as the corresponding parental bacteria (Table 1; Table S3). We found that whereas the bacteria bound to many types of mono- and di-saccharides, this was not the case for EVs; only those from the strain 81-176 were able to bind any of the simple sugars (Table 1). By contrast, EVs showed binding preferences for complex glycans, such as Lewis antigens, blood group A antigens, and gangliosides. Importantly, EVs were shown to share receptor binding specificities with *C. jejuni* bacteria for fucosyl GM1 ganglioside, P1 blood group antigen, sialyl Lewis^x^ (Le^x^), and sulfated Le^x^ (Table 1). Importantly, these interactions were observed for three different *C. jejuni* strains. Together, these data provide the first evidence that EVs share host receptor specificities with the bacteria and, moreover, that complex glycans may be involved in EV interactions and uptake in epithelial cells.

### EV-associated *C. jejuni* CDT induces cell cycle arrest and cell distension in HeLa cells

**Fig 4:**
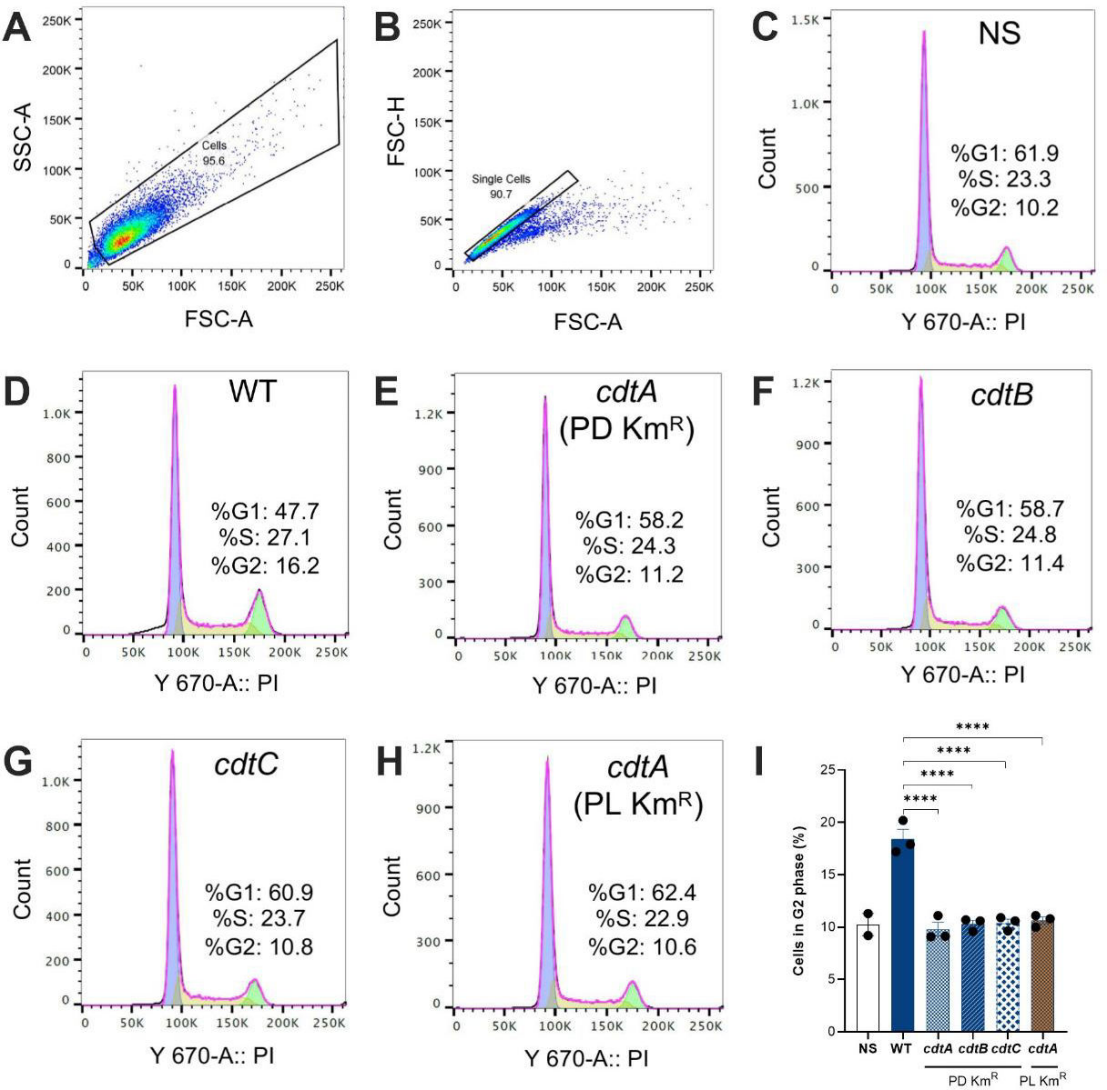
WT EVs but not those from *cdt* mutants induce cell cycle arrest in HeLa cells. EVs (5 µg/mL) from *C. jejuni* 81-176 WT or *cdt* mutant bacteria were added to HeLa cells and incubated for 24 hours before staining with propidium iodide (PI) and analysis by flow cytometry. (**A, B**) The gating strategy used for flow cytometry. (**C-H**) Histograms showing the numbers of cells undergoing G1 (purple), S (yellow), and G2 (green) phases. Control cells were not exposed to EVs (not stimulated, NS). (**I**) Quantification of cells in the G2 phase from each treatment group. Data represent the means ± SEM for two or three biological replicates, as indicated. Groups were analyzed using one-way ANOVA and the Tukey multiple comparison test. ****P < 0.0001. NS, non-stimulated.

*C. jejuni* culture supernatants or cell lysates are known to induce cell cycle arrest at the G2/M phase and cell distension in susceptible eucaryotic cells (4, 24–26). Interestingly, it was reported that the majority of extracellular CDT in *C. jejuni* culture supernatants was associated with EVs (18). Furthermore, EVs were reported to contain all three CDT subunits and can reproduce the cell distension in human intestinal cells, similar to that induced by the toxin (18). We sought to extend these studies by assessing the induction of cell cycle arrest and cell distension in HeLa cells incubated with EVs from either *C. jejuni* WT, *cdtA, cdtB,* or *cdtC* mutant bacteria. Cell cycle arrest was measured by flow cytometry (Fig. 4A and B) which revealed a significant increase in the mean numbers of cells in the G2 phase for cells incubated with EVs from WT *C. jejuni* when compared with those exposed to any of the *cdt* mutant bacteria (18.4% versus 9.7%–10.6%, respectively; *P <* 0.001) (Fig. 4C through I). The lack of activity for *cdt* mutant EVs can be attributed to aberrant *cdt* gene expression (Fig. S2A through S2D) and protein production (Fig. S2E through S2G), resulting in the absence of the CdtB subunit, which is responsible for DNA damage and cell cycle arrest. This did not appear to be due to potential polar effects from the PD Km^R^ cassette on downstream genes, as a *cdtA* mutant constructed using the PL Km^R^ cassette had WT levels of *cdt* gene expression (Fig. S2D) but did not produce any of the subunits (Fig. S2G), suggesting that destabilization of the expression of any individual gene impacts production of the Cj-CDT holotoxin.

**Fig 5:**
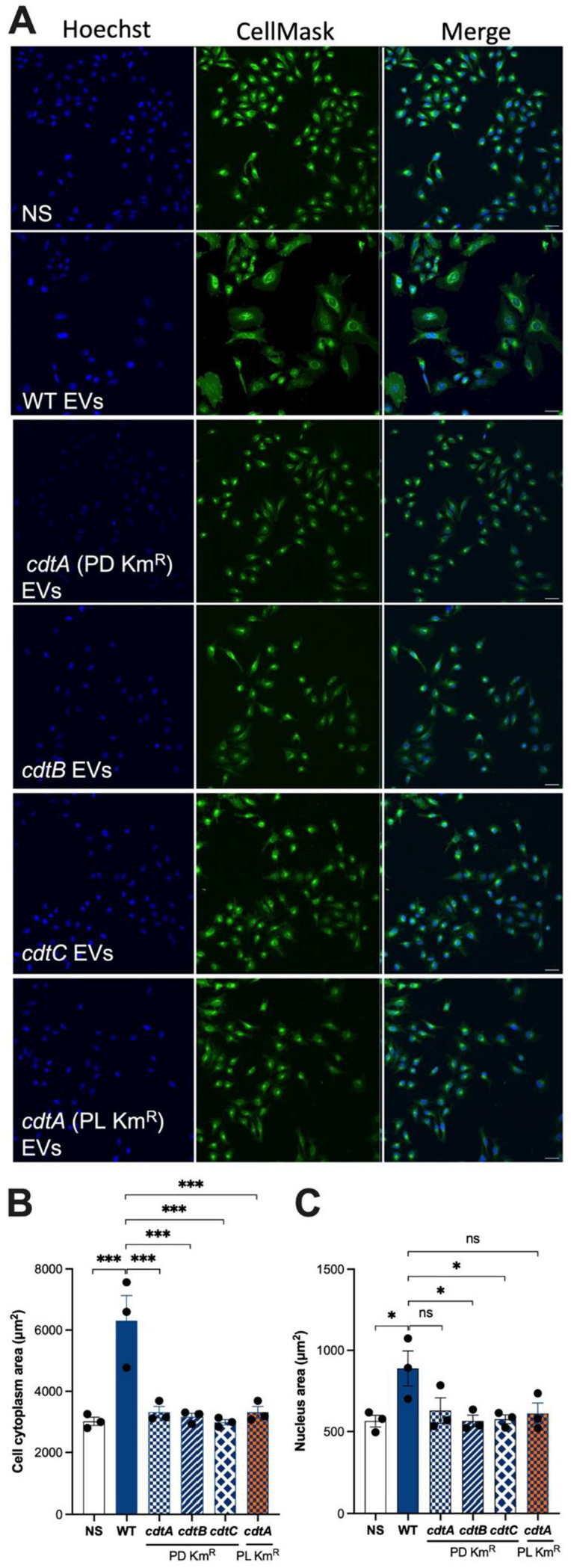
WT EVs but not those from *cdt* mutants induced cell distension in HeLa cells. EVs (5 µg/mL) from *C. jejuni* 81-176 WT or *cdt* mutant bacteria were added to HeLa cells and incubated for 48 hours before staining with Hoechst (nucleus) and CellMask Green (plasma membrane) dyes. Control cells were not exposed to EVs (not stimulated, NS). (**A**) Cells were imaged using an Olympus FV1200 Confocal Microscope (20× magnification). Scale bar = 50 µm. (**B**) The cell cytoplasm and (**C**) nucleus areas in individual cells were quantified using Imaris software. Data represent the means ± SEM for three biological replicates. Groups were analyzed using one-way ANOVA and the Tukey multiple comparison test. NS, not significant, *P < 0.05, ****P < 0.001.

Consistent with the cell cycle arrest data for EV-associated CDT (Fig. 4), we observed that cells exposed to WT EVs induced significantly more cell distension, as measured by increased cytoplasm and nucleus areas when compared with control cells (*P <* 0.001) (Fig. 5A and C). Furthermore, the cytoplasm and nuclei of cells that had been incubated with WT EVs were nearly two times larger than those exposed to EVs from any of the *cdt* mutants (Fig. 5B and C). The results demonstrated that Cj-CDT holotoxin is required for EV induction of cell cycle arrest and cell distension, two key characteristics of all members of the CDT family.

## DISCUSSION

*C. jejuni* is a major cause of food-borne gastroenteritis worldwide; yet, the molecular basis behind its pathogenesis has not been fully elucidated. CDT is an important virulence factor of *C. jejuni*; however, this toxin is the least characterized member of the CDT family. Although one study found that the majority of secreted Cj-CDT was associated with EVs (18), there have not been any follow-up studies on this topic. Here, we have characterized the CDT that is associated with *C. jejuni* EVs and determined its effects on host cells. Importantly, we have shown that CdtAC subunits, which are the cell surface-binding moieties of the toxin and facilitate translocation of the active CdtB subunit into host cells (4), are not required for EV binding to cells. Moreover, we have identified complex host cell glycans as potential receptors for EV binding and uptake in epithelial cells.

To better understand the role of EV-associated Cj-CDT in *C. jejuni* pathogenesis, we generated *cdtA*, *cdtB,* and *cdtC* mutants by double homologous recombination. Aberrant *cdt* gene expression was observed in the mutants generated using the PD Km^R^ cassette (Fig. S2A through S2C). This could be explained by the fact that the Cj-CDT holotoxin is encoded on an operon, in which *cdtA* overlaps *cdtB* by four base pairs (bp), whereas *cdtB* and *cdtC* are 10 bp apart (43). Nevertheless, the *cdtA* mutant, which was constructed with an intact ribosomal binding site and in-frame insertion of the PL Km^R^ cassette, expressed similar levels of *cdtB* and *cdtC* as the WT strain (Fig. S2D), yet did not produce any of the subunits (Fig. S2G). This result may be explained by changes in mRNA folding or stability which are known to potentially hinder the translation initiation of contiguous genes or decrease their translational coupling (44, 45). One explanation may be that all three CDT subunits are needed for toxin stability, with the lack of one subunit resulting in the other subunits becoming unstable and leading to their degradation. Further studies are warranted to understand the regulation of the *C. jejuni* CDT operon.

Despite the aberrant expression and production of CDT subunits observed in the mutant *C. jejuni* strains, we did not find any differences in EV morphologies, size distributions, or vesicle numbers between WT and mutant bacteria (Fig. 1). EV sizes for the *C. jejuni* WT and mutant bacteria ranged from 5 to 530 nm which, however, differed from the reported sizes of 10–50 nm (18) or 10–250 nm (20) in previous studies. These observed differences may be attributed to the fact that NTA was used to measure EV sizes in the current work, whereas previous studies used TEM. Significantly, we demonstrated that EVs from WT *C. jejuni* bacteria, but not those from any of the *cdt* mutant bacteria, were able to induce cell cycle arrest (Fig. 4) and cell distension (Fig. 5) in epithelial cells. This is consistent with the absence of CdtB production by the mutant bacteria (Fig. S2E through S2G).

Although CDTs have been associated with EVs (18, 20), the cellular localization of these toxins is unknown. Immunoblotting of PK-treated *C. jejuni* EVs with anti-CDT sera showed that Cj-CDT is predominantly localized within EVs (Fig. 2), suggesting that it is protected within EVs from external enzymes or other factors. This conclusion, however, does raise questions regarding the established functions of soluble CdtAC as the binding subunits for the toxin (46). The absence of CdtA and CdtC from the surfaces of EV suggests that these subunits most likely do not participate in the binding and entry of EVs into host cells despite both these two subunits and EVs being reported to bind lipid rafts (47, 48). It is important to note, however, that most of the scientific literature on CDTs has arisen using either recombinant CDT subunits or cell lysates containing CDT. It is possible that EV-associated CDT subunits have different functions to those of soluble or membrane-bound proteins, as reported for vacuolating cytotoxin A from *Helicobacter pylori* (49). Consistent with this suggestion, a study by reference (50) examined the intracellular trafficking of the CDT produced by enterohemorrhagic *E. coli* (EHEC) and found that once inside host cells, CdtB separates from EV-associated CdtAC (50). CdtB then traffics to the nucleus and induces cell cycle arrest, whereas the CdtAC subunits traffic to the lysosome for degradation (50). This suggests that EV-associated CdtAC subunits from EHEC may be redundant. Whether this is also the case for *C. jejuni* CdtAC requires further investigation.

An important observation from the current work was that PK-treated EVs, in which surface proteins were cleaved, could still induce CdtB-associated cell distension to similar levels as untreated EVs (Fig. 3). Interestingly, another study also observed that PK-treated *C. jejuni* EVs induced similar interleukin-8 (IL-8) and human β-defensin-3 (hβD-3) levels, as cells stimulated with untreated EVs (20). Together, these data show that important virulence factors are protected inside EVs and that, furthermore, EVs lacking surface proteins were able to enter epithelial cells and induce a range of responses. Thus, classical protein-based ligands may not be required for receptor interactions with host cells and the uptake of EVs. One study found that *C. jejuni* EVs interact with host cells *via* lipid rafts, which are enriched in cholesterol, glycosphingolipids, and sphingolipids (20). The cellular receptor(s) within lipid rafts that are responsible for *C. jejuni* EV binding and uptake into host cells remain(s) to be elucidated. In the current study, *C. jejuni* EVs were shown for the first time to bind complex host glycans and have shared binding specificities with *C. jejuni* bacteria for fucosyl GM1 ganglioside, P1 blood group antigen, sialyl Lewis^x^ (Le^x^), and sulfated Le^x^ (Table 1). Interestingly, GM1 and P1 blood group antigens are known to serve as receptors for cholera (51) and Shiga toxin (52), respectively. Further studies are therefore warranted to confirm whether these host glycans may be receptors responsible for the entry of *C. jejuni* EVs into host cells.

Another important question requiring further investigation is the role of non-protein ligands in the binding of *C. jejuni* EVs to host cells. Day *et al.* reported that bacterial glycans can form high-affinity interactions with host glycans to mediate their attachment to host tissues (53). Most importantly, glycan array analysis revealed lipooligosaccharide (LOS)/lipopolysaccharide (LPS) from various bacterial pathogens, including *C. jejuni*, to bind numerous host glycan structures (53). Whether EV-associated LOS/LPS mediates EV entry into host cells by binding to host glycans requires further investigation. Furthermore, it will be important to determine whether *N*-glycans are required for CDT-mediated cell toxicity by EVs, as reported previously (8). As CDTs and EVs are increasingly considered to be promising candidates for therapeutic applications, including as anti-tumor therapies, it is important to gain a better understanding of the biology of EV-associated CDTs.

## SUPPLEMENTAL MATERIAL

Supplemental material spectrum.03232-23-s0001.docx Tables S1 to S3; Figures S1 and S2.

ASM does not own the copyrights to Supplemental Material that may be linked to, or accessed through, an article. The authors have granted ASM a non-exclusive, world-wide license to publish the Supplemental Material files. Please contact the corresponding author directly for reuse.
