# Supplementary material for: Campylobacter jejuni extracellular vesicles harboring cytolethal distending toxin bind host cell glycans and induce cell cycle arrest in host cells
Source: Microbiol Spectr. 2024 Feb 6;12(3):e03232-23. doi: 10.1128/spectrum.03232-23 (PMC10913475; doi:10.1128/spectrum.03232-23)
Supplement: Supplemental material — Tables S1 to S3; Figures S1 and S2. [file spectrum.03232-23-s0001.docx]

**Table S1. A list of primers used in this study.**

| Primer name | Primer sequence | Source |
| --- | --- | --- |
| cdtA-For | 5’- TAAATCCTTTGGGGCGTTC -3’ | (25) |
| cdtA-Rev | 5’- TCATCGTACCTCTCCTTGGC -3’ | This paper |
| cdtA-BamHI | 5’- CGGGATCCACCAATACTTGTCTTAATGC -3’ | This paper |
| cdtA-KnpI | 5’- GGGGTACCAACCGTTAAAGCTGCTCC -3’ | This paper |
| H17 | 5’- TTTGACTTACTGGGGATCAAGCCTG -3’ | (48) |
| H50 | 5’- CCGGTGATATTCTCATTTTAGCC -3’ | (48) |
| cdtB-For | 5’- GAATTTGCAAGGCTCATCC -3’ | This paper |
| cdtB-Rev | 5’- CAAGCATTAAAATCGCAGC -3’ | This paper |
| cdtC-For | 5’- GCCTTTGCAACTCCTACTG -3’ | (25) |
| cdtC-Rev | 5’- AGGGGTAGCAGCTGTTAAAG -3’ | This paper |
| 16s-For | 5’- GGAGTACGGTCGCAAGATTAAA -3’ | (49) |
| 16s-Rev | 5’- ACTTCACCCCAGTCGCTG -3’ | (50) |

| **Antibody name** | **Bacteria species** | **Dilution** | **Source** |
| --- | --- | --- | --- |
| Rabbit anti-CdtA serum | *C. jejuni* | 1:2500 | (25) |
| Rabbit anti-CdtB serum | *C. jejuni* | 1:2500 | (25) |
| Rabbit anti-CdtC serum | *C. jejuni* | 1:2500 | (25) |
| Rabbit anti-EV serum | *C. jejuni* | 1:10,000 | This paper |
| Rabbit anti-GroEL/HspB serum | *H. pylori* | 1:1000 | (51) |
| Mouse anti-RNA sigma 70/ RpoD antibody (clone 2G10) | *E. coli* | 1:1000 | Biolegend, catalog no. 663207 |

**Table S2. A list of antibodies used in this study.**

**Table S3. The full data set of glycans bound by bacteria and EVs of *C. jejuni* strains 81116, 81-176 and 11168.**

| Glycan type | Strains | | | | | |
| --- | --- | --- | --- | --- | --- | --- |
|  | 81116 | 81116 -OMV | 81-176 | 81-176 -OMV | 11168 | 11168-OMV |
| Terminal galactose | - Galβ1-3GlcNAc - Galβ1-4GlcNAc - Galβ1-4Gal - GalNAcα1-O-Ser - Galα1-4Galβ1-4Glc - Galα1-3Gal - Galβ1-4GlcNAcβ1-3Galβ1-4Glc - Galβ1-4GlcNAcβ1-6(Galβ1-4GlcNAcβ1-3)Galβ1-4Glc - Galβ1-4GlcNAcβ1-6(Galβ1-3GlcNAcβ1-3)Galβ1-4Glc - Galα1-3Galβ1-4Glc - Galα1-3Galβ1-4Galα1-3Gal - GalNAcβ1-3Gal - Galα1-4Galβ1-4GlcNAc - GalNAcα1-3Galβ1-4Glc - Galβ1-3GlcNAcβ1-3Galβ1-4GlcNAcβ1-6(Galβ1-3GlcNAcβ1-3)Galβ1-4Glc | - | - Galβ1-3GlcNAc - Galβ1-4GlcNAc - Galβ1-4Gal - Galα1-3Gal - Galα1-3Galβ1-4Glc - Galβ1-3GlcNAcβ1-3Galβ1-4Glc - Galβ1-4GlcNAcβ1-6(Galβ1-4GlcNAcβ1-3)Galβ1-4Glc - Galβ1-4GlcNAcβ1-6(Galβ1-3GlcNAcβ1-3)Galβ1-4Glc - Galα1-3Galβ1-4Galα1-3Gal - GalNAcβ1-3Gal - Galα1-4Galβ1-4GlcNAc - GalNAcα1-3Galβ1-4Glc - Galβ1-3GlcNAcβ1-3Galβ1-4GlcNAcβ1-6(Galβ1-3GlcNAcβ1-3)Galβ1-4Glc - Galβ1-3GalNAc | - | - Galβ1-3GlcNAc - Galβ1-4GlcNAc - Galβ1-4Gal - Galβ1-6GlcNAc - Galα1-4Galβ1-4Glc - GalNAcα1-O-Ser - Galβ1-3GalNAcα1-O-Ser - Galβ1-4Gal - GalNAcα1-3Galβ1-4Glc - Galβ1-4GlcNAcβ1-6(Galβ1-3GlcNAcβ1-3)Galβ1-4Glc - Galβ1-3GlcNAcβ1-3Galβ1-4GlcNAcβ1-6(Galβ1-3GlcNAcβ1-3)Galβ1-4Glc - Galα1-3Galβ1-4Galα1-3Gal - GalNAcβ1-3Gal - Galα1-4Galβ1-4GlcNAc | - |
| Terminal GlcNAc | - GlcNAcβ1-4GlcNAc - GlcNAcβ1-4GlcNAcβ1-4GlcNAc - GlcNAcβ1-4GlcNAcβ1-4GlcNAcβ1-4GlcNAcβ1-4GlcNAc - GlcNAcβ1-4GlcNAcβ1-4GlcNAcβ1-4GlcNAcβ1-4GlcNAcβ1-4GlcNAc - GlcNAcβ1-4GlcNAcβ1-4GlcNAcβ1-4GlcNAc | - GlcNAcβ1-4GlcNAcβ1-4GlcNAcβ1-4GlcNAcβ1-4GlcNAc - GlcNAcβ1-4GlcNAcβ1-4GlcNAcβ1-4GlcNAcβ1-4GlcNAcβ1-4GlcNAc - GlcNAcβ1-4MurNAc | - GlcNAcβ1-4GlcNAcβ1-4GlcNAcβ1-4GlcNAcβ1-4GlcNAcβ1-4GlcNAc - GlcNAcβ1-4MurNAc - GlcNAcβ1-4GlcNAcβ1-4GlcNAcβ1-4GlcNAc | - GlcNAcβ1-4MurNAc | - GlcNAcβ1-4GlcNAcβ1-4GlcNAc - GlcNAcβ1-4GlcNAc - GlcNAcβ1-4GlcNAcβ1-4GlcNAcβ1-4GlcNAc - GlcNAcβ1-4GlcNAcβ1-4GlcNAcβ1-4GlcNAcβ1-4GlcNAcβ1-4GlcNAc | - GlcNAcβ1-4GlcNAcβ1-4GlcNAc - GlcNAcβ1-4GlcNAc - GlcNAcβ1-4MurNAc - GlcNAcβ1-4GlcNAcβ1-4GlcNAcβ1-4GlcNAcβ1-4GlcNAc |
| Mannosyl containing glycans | - Manα1-2Man - Manα1-4Man - Manα1-6(Manα1-3)Manα1-6(Manα1-3)Man - Manα1-6(Manα1-3)Man - GlcNAcβ1-2Man - GlcNAcβ1-2Manα1-6(GlcNAcβ1-2Manα1-3)Man | - Manα1-4Man | - Manα1-2Man - Manα1-6(Manα1-3)Manα1-6(Manα1-3)Man - Manα1-4Man - Manα1-6(Manα1-3)Man - GlcNAcβ1-2Man | - Manα1-2Man | - Manα1-2Man - Manα1-4Man - Manα1-6Man - Manα1-6(Manα1-3)Manα1-6(Manα1-3)Man - Manα1-6(Manα1-3)Man - GlcNAcβ1-2Man - GlcNAcβ1-2Manα1-6(GlcNAcβ1-2Manα1-3)Man | - Manα1-4Man - Manα1-6Man |
| Fucosylated glycans | - Fucα1-2Galβ1-3(Fucα1-4)GlcNAcβ1-3Gal (Le^b^) - Galβ1-3(Fucα1-4)GlcNAc (Le^a^) - Fucα1-2Galβ1-4(Fucα1-3)Glc - Galβ1-4(Fucα1-3)GlcNAcβ1-3Galβ1-4Glc - Fucα1-2Galβ1-3(Fucα1-4)GlcNAcβ1-3Galβ1-4Glc - Galβ1-3(Fucα1-4)GlcNAcβ1-3Galβ1-4(Fucα1-3)Glc - Fucα1-2Gal - Fucα1-2Galβ1-3GlcNAcβ1-3Galβ1-4Glc - GalNAcα1-3(Fucα1-2)Galβ1-3GalNAcβ1-3Gal (Blood group A antigen pentaose type 4) - Galβ1-3GlcNAcβ1-3Galβ1-4(Fucα1-3)GlcNAcβ1-3Galβ1-4Glc - GalNAcα1-3(Fucα1-2)Galβ1-4GalNAc - Galβ1-4(Fucα1-3)GlcNAcβ1-6(Galβ1-3GlcNAcβ1-3)Galβ1-4Glc   - Galβ1-4(Fucα1-3)GlcNAcβ1-6(Fucα1-2Galβ1-3GlcNAcβ1-3)Galβ1-4Glc | - Galβ1-3(Fucα1-4)GlcNAc (Le^a^) - Fucα1-2Galβ1-4(Fucα1-3)Glc   -  -  -  -  -  -  -  -  -  -   - GalNAcα1-3(Fucα1-2)Galβ1-3GalNAcβ1-3Gal (Blood group A antigen pentaose type 4) | - Fucα1-2Galβ1-3(Fucα1-4)GlcNAcβ1-3Gal (Le^b^) - Fucα1-2Galβ1-4(Fucα1-3)Glc - SO3-3Galβ1-4(Fucα1-3)GlcNAc (Le^x^) - Galβ1-3GlcNAcβ1-3Galβ1-4(Fucα1-3)GlcNAcβ1-3Galβ1-4Glc - Galβ1-4(Fucα1-3)GlcNAcβ1-6(Galβ1-3GlcNAcβ1-3)Galβ1-4Glc - Galβ1-4(Fucα1-3)GlcNAcβ1-6(Fucα1-2Galβ1-3GlcNAcβ1-3)Galβ1-4Glc - GalNAcα1-3(Fucα1-2)Galβ1-3GalNAcβ1-3Gal (Blood group A antigen pentaose type 4) - GalNAcα1-3(Fucα1-2)Galβ1-4GalNAc - Galβ1-4(Fucα1-3)GlcNAcβ1-3Galβ1-4Glc - Fucα1-2Galβ1-3(Fucα1-4)GlcNAcβ1-3Galβ1-4Glc - Galβ1-3(Fucα1-4)GlcNAcβ1-3Galβ1-4(Fucα1-3)Glc | -  -  -  -  -  -  -  -  -  -  -  -   - GalNAcα1-3(Fucα1-2)Galβ1-3GalNAcβ1-3Gal (Blood group A antigen pentaose type 4) | - Fucα1-2Galβ1-3(Fucα1-4)GlcNAcβ1-3Gal (Le^b^) - Fucα1-2Galβ1-3GlcNAcβ1-3Galβ1-4Glc - Galβ1-4(Fucα1-3)GlcNAcβ1-3Galβ1-4Glc - Galβ1-3(Fucα1-4)GlcNAcβ1-3Galβ1-4(Fucα1-3)Glc - Galα1-3(Fucα1-2)Galβ1-3GalNAcβ1-3Gal (Blood group B antigen pentasaccaride type 4) - Galα1-3(Fucα1-2)Galβ1-4Glc (Blood group B antigen tetraose type 5) - GalNAcα1-3(Fucα1-2)Galβ1-4(Fucα1-3)Glc (Blood group A pentasaccharide) - Fucα1-2Galβ1-4Glc - Fucα1-2Galβ1-4(Fucα1-3)GlcNAcβ1-3Galβ1-4Glc - Galβ1-3GlcNAcβ1-3Galβ1-4(Fucα1-3)GlcNAcβ1-3Galβ1-4Glc - Galβ1-4(Fucα1-3)GlcNAcβ1-6(Fucα1-2Galβ1-3GlcNAcβ1-3)Galβ1-4Glc | - Galα1-3(Fucα1-2)Galβ1-4(Fucα1-3)Glc (Blood Group B pentasaccharide) - Galα1-3(Fucα1-2)Galβ1-3GalNAcβ1-3Gal (Blood group B antigen pentasaccaride type 4) - GalNAcα1-3(Fucα1-2)Galβ1-4(Fucα1-3)Glc (Blood group A pentasaccharide) |
| Sialylated glycans | - Neu5Acα2-3Galβ1-3GlcNAcβ1-3Galβ1-4Glc - Neu5Acα2-3Galβ1-4GlcNAc - Neu5Acα2-3Galβ1-3(Fucα1-4)GlcNAc (Sia-Le^a^) - Galβ1-3GlcNAcβ1-3(Neu5Acα2-6Galβ1-4GlcNAcβ1-6)Galβ1-4Glc - Neu5Acα2-6Galβ1-3GlcNAcβ1-3(Galβ1-4GlcNAcβ1-6)Galβ1-4Glc - Neu5Acα2-3Galβ1-4(Fucα1-3)GlcNAc ( S Le^x^) - Galβ1-4(Fucα1-3)GlcNAcβ1-6(Neu5Acα2-6Galβ1-4GlcNAcβ1-3)Galβ1-4Glc - Galβ1-3(Neu5Acα2-6)GlcNAcβ1-3Galβ1-4Glc - Neu5Acα2-6Galβ1-4GlcNAcβ1-3Galβ1-4Glc - Neu5Acα2-6Galβ1-4GlcNAc - Neu5Acα2-3Galβ1-3(Neu5Acα2-6)GlcNAcβ1-3Galβ1-4Glc | - Neu5Acα2-3Galβ1-3GlcNAcβ1-3Galβ1-4Glc - Neu5Acα2-3Galβ1-4GlcNAc | - Neu5Acα2-3Galβ1-3GlcNAcβ1-3Galβ1-4Glc - Neu5Acα2-3Galβ1-4GlcNAc - Neu5Acα2-3Galβ1-3(Fucα1-4)GlcNAc (Sia-Le^a^) - Neu5Acα2-3Galβ1-3(Neu5Acα2-6)GlcNAcβ1-3Galβ1-4Glc - Galβ1-3GlcNAcβ1-3(Neu5Acα2-6Galβ1-4GlcNAcβ1-6)Galβ1-4Glc - Neu5Acα2-6Galβ1-3GlcNAcβ1-3(Galβ1-4GlcNAcβ1-6)Galβ1-4Glc - Neu5Acα2-6Galβ1-4GlcNAcβ1-3Galβ1-4Glc - Neu5Acα2-3Galβ1-4Glc - Galβ1-4(Fucα1-3)GlcNAcβ1-6(Neu5Acα2-6Galβ1-4GlcNAcβ1-3)Galβ1-4Glc - Neu5Acα2-3Galβ1-3(Neu5Acα2-6)GalNAc - Neu5Acα2-6Galβ1-3GlcNAcβ1-3Galβ1-4(Fucα1-3)Glc - Galβ1-3(Neu5Acα2-6)GlcNAcβ1-3Galβ1-4Glc | - Neu5Acα2-3Galβ1-3GlcNAcβ1-3Galβ1-4Glc - Neu5Acα2-3Galβ1-3(Fucα1-4)GlcNAc (Sia-Le^a^) - Neu5Acα2-3Galβ1-3(Neu5Acα2-6)GlcNAcβ1-3Galβ1-4Glc | - Neu5Acα2-3Galβ1-3GlcNAcβ1-3Galβ1-4Glc - Neu5Acα2-3Galβ1-4GlcNAc - Neu5Acα2-3Galβ1-4(Fucα1-3)GlcNAc ( S Le^x^) - Neu5Acα2-3Galβ1-3(Neu5Acα2-6)GlcNAcβ1-3Galβ1-4Glc - Neu5Acα2-3Galβ1-3(Fucα1-4)GlcNAc(Sia-Le^a^) - Neu5Acα2-6Galβ1-4GlcNAcβ1-3Galβ1-4Glc - Neu5Acα2-6Galβ1-3GlcNAcβ1-3(Galβ1-4GlcNAcβ1-6)Galβ1-4Glc - Galβ1-4(Fucα1-3)GlcNAcβ1-6(Neu5Acα2-6Galβ1-4GlcNAcβ1-3)Galβ1-4Glc - Neu5Acα2-6Galβ1-4GlcNAcβ1-3Galβ1-4Glc | - Neu5Acα2-3Galβ1-3GlcNAcβ1-3Galβ1-4Glc - Neu5Acα2-3Galβ1-4(Fucα1-3)GlcNAc( S Le^x^) - Neu5Acα2- 3Galβ1-3(Neu5Acα2-6)GlcNAcβ1-3Galβ1-4Glc - Neu5Acα2-3Galβ1-3(Fucα1-4)GlcNAc(Sia-Le^a^) |
| Ganglioside structures | - Neu5Acα2-8Neu5Acα2-3Galβ1-3GalNAcβ1-4(Neu5Acα2-3)Galβ1-4Glc (GT1a) - Fucα1-2Galβ1-3GalNAcβ1-4(Neu5Acα2-3)Galβ1-4Glc (fucosyl GM1) - Galβ1-3GalNAcβ1-4Galβ1-4Glc - Galβ1-3GalNAcβ1-4(Neu5Acα2-8Neu5Acα2-8 Neu5Acα2-3)Galβ1-4Glc - Neu5Acα2-8Neu5Acβ2-8Neu5Acα2-3Galβ1-4Glc | - Neu5Acα2-8Neu5Acα2-3Galβ1-3GalNAcβ1-4(Neu5Acα2-3)Galβ1-4Glc (GT1a) - Fucα1-2Galβ1-3GalNAcβ1-4(Neu5Acα2-3)Galβ1-4Glc (fucosyl GM1)   -  -   - Neu5Acα2-8Neu5Acα2-3Galβ1-4Glc (GD3) | - Neu5Acα2-8Neu5Acβ2-8Neu5Acα2-3Galβ1-4Glc (GT3) - Fucα1-2Galβ1-3GalNAcβ1-4(Neu5Acα2-3)Galβ1-4Glc (fucosyl GM1) - Galβ1-3GalNAcβ1-4Galβ1-4Glc - Galβ1-3GalNAcβ1-4(Neu5Acα2-8Neu5Acα2-8 Neu5Acα2-3)Galβ1-4Glc - Neu5Acα2-3Galβ1-3GalNAcβ1-4(Neu5Acα2-3)Galβ1-4Glc  (GD1a) | - Neu5Acα2-8Neu5Acβ2-8Neu5Acα2-3Galβ1-4Glc (GT3) - Fucα1-2Galβ1-3GalNAcβ1-4(Neu5Acα2-3)Galβ1-4Glc (fucosyl GM1) - Neu5Acα2-3Galβ1-3GalNAcβ1-4(Neu5Acα2-3)Galβ1-4Glc (GD1a) | - Galβ1-3GalNAcβ1-4(Neu5Acα2-8Neu5Acα2-8 Neu5Acα2-3)Galβ1-4Glc (GT1c) - Fucα1-2Galβ1-3GalNAcβ1-4(Neu5Acα2-3)Galβ1-4Glc (fucosyl GM1) - Neu5Acα2-8Neu5Acα2-3Galβ1-3GalNAcβ1-4(Neu5Acα2-3)Galβ1-4Glc (GT1a) - Galβ1-3GalNAcβ1-4(Neu5Acα2-8Neu5Acα2-3)Galβ1-4Glc (GD1b ganglioside sugar) - Neu5Acα2-3Galβ1-3GalNAcβ1-4(Neu5Acα2-3)Galβ1-4Glc (GD1a) | - Neu5Acα2-3Galβ1-3GalNAcβ1-4(Neu5Acα2-3)Galβ1-4Glc (GD1a) - Fucα1-2Galβ1-3GalNAcβ1-4(Neu5Acα2-3)Galβ1-4Glc (fucosyl GM1) - Galβ1-3GalNAcβ1-4(Neu5Acα2-8Neu5Acα2-8 Neu5Acα2-3)Galβ1-4Glc (GT1c) |

**
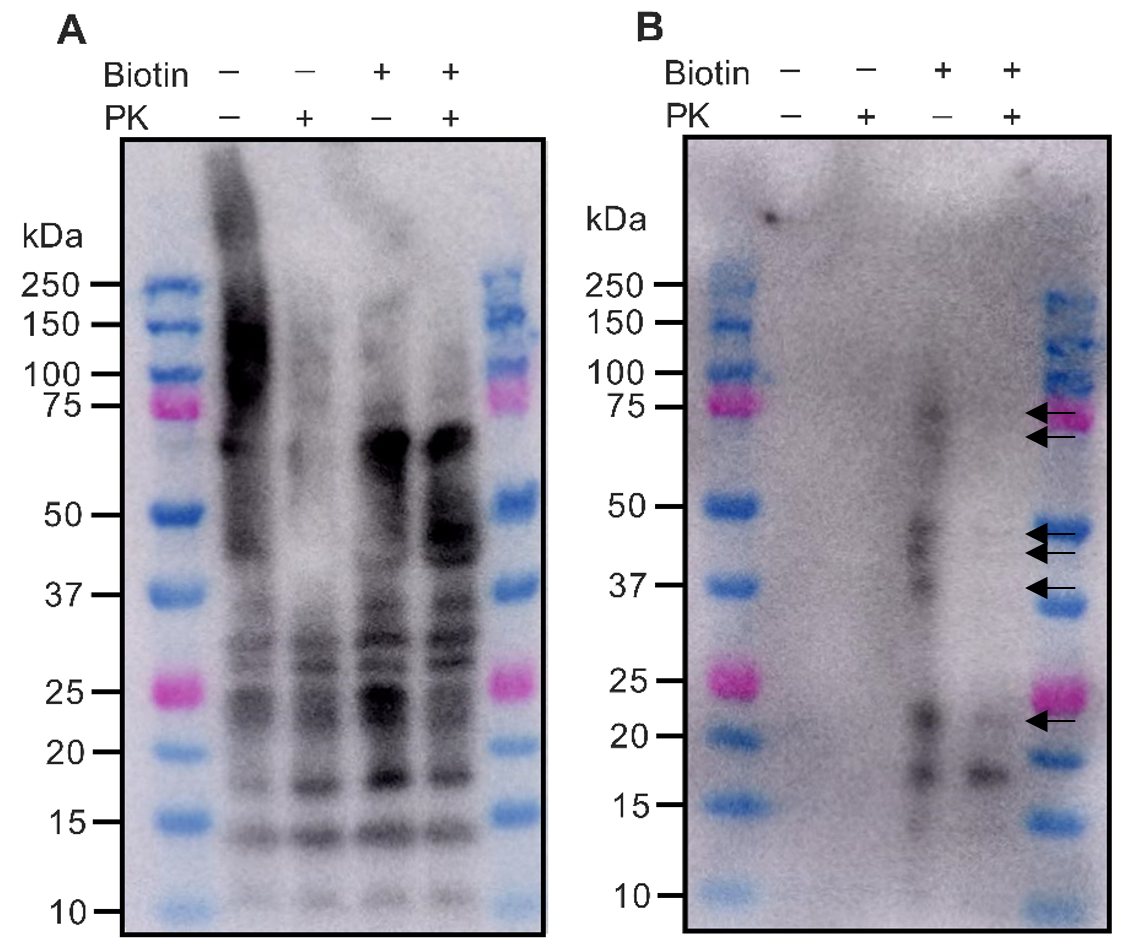
**

**Figure S1.** Proteinase K digestion of surface-accessible proteins on *C. jejuni* EVs. *C. jejuni* EVs were labelled with DSB-X biotin then treated with proteinase K (PK) before samples were analyzed by immunoblotting with: (A) anti-*C. jejuni* EV serum; or (B) streptavidin-horseradish peroxidase (HRP). Arrows indicate putative surface-accessible proteins that had been digested. Immunoblots are from one independent experiment.


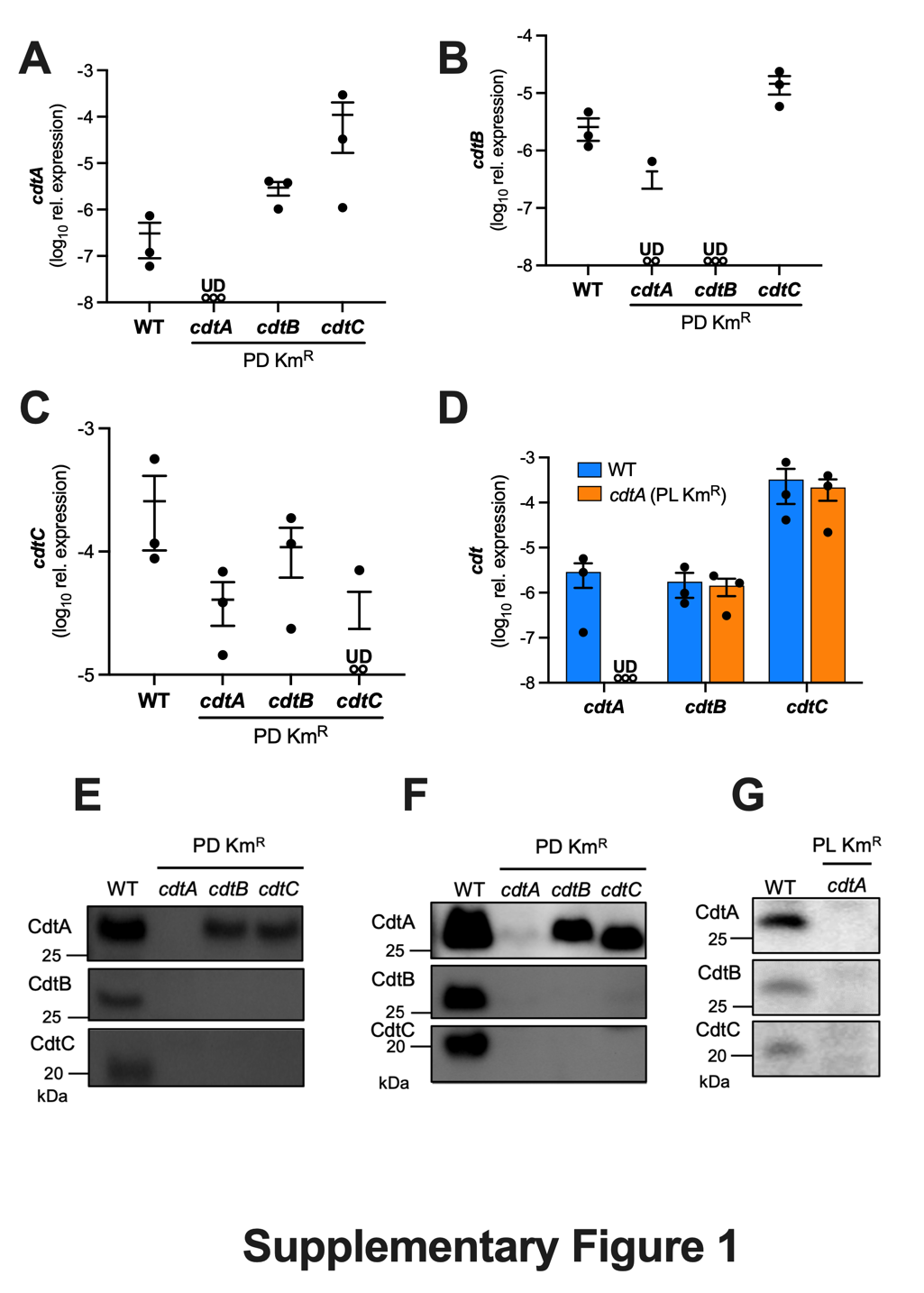


**Figure S2. Characterization of *cdt* expression and CDT subunit production in *C. jejuni* WT and *cdt* mutant bacteria.** *C. jejuni* 81-176 WT and *cdt* mutants generated using the PD Km^R^ cassette were analyzed for (A) *cdtA*, (B) *cdtB* and (C) *cdtC* gene expression by qPCR. (D) *cdt* gene expression for *C. jejuni* 11168-O WT and *cdtA* mutant generated using the PL Km^R^ cassette. Gene expression was normalized to 16S rRNA. Data represent the means ± SEM for three biological replicates. (E, G) Whole cell lysates and (F) EVs isolated from *C. jejuni* 81-176 WT and *cdt* mutants were immunoblotted (7.5 µg) against anti-CdtA, CdtB and CdtC sera. Representative images of two independent experiments.
